# Plantar Lichen Planus: An Atypical Presentation at a Young Age

**DOI:** 10.7759/cureus.17851

**Published:** 2021-09-09

**Authors:** Paras Sisodia, Shubham Tripathi, Parul Verma, Atin Singhai

**Affiliations:** 1 Internal Medicine, King George's Medical University, Lucknow, IND; 2 Dermatology, King George's Medical University, Lucknow, IND; 3 Pathology, King George's Medical University, Lucknow, IND

**Keywords:** lichen planus, plantar lichen planus, foot dermatoses, erythematous plaques, atypical lichen planus

## Abstract

Lichen planus is a chronic lichenoid dermatosis commonly encountered by dermatologists worldwide, affecting skin, mucosa, and scalp. The current case describes a rare variant of lichen planus, plantar lichen planus, in a 17-year-old male who presented with erythematous scaly plaques on the sole for two years associated with walking discomfort. The lesion was subjected to skin biopsy and a diagnosis of lichen planus was made considering the histopathological and clinical findings. Plantar lichen planus can often be misdiagnosed. Treating plantar lichen planus can be a therapeutic challenge and, thus, more insight is needed regarding treatment protocol or outcome of such cases.

## Introduction

Lichen planus (LP) is a chronic characteristic lichenoid dermatosis of the skin associated with relapses and remission. It is a T-cell-mediated destruction of keratinocytes often triggered by drugs, allergens, and viruses [1]. Lesions of classic LP are polygonal papules that are red to violet in color and are often associated with pruritus, which may range from mild irritation to severe itching [1]. Multiple variants of LP have been described in the literature like hypertrophic LP, annular LP, actinic LP, bullous LP, linear LP, and follicular LP [2,3].

A rare clinical variant of LP is the palmoplantar LP (PPLP), which has been described less frequently in the literature, and cases of palmoplantar LP are often misdiagnosed as psoriasis, eczema, tinea manuum infection, etc [4]. We here describe an unusual case of plantar LP in a 17-year-old male who presented with erythematous plaques on the left sole for two years associated with walking discomfort.

## Case presentation

**Figure 1:**
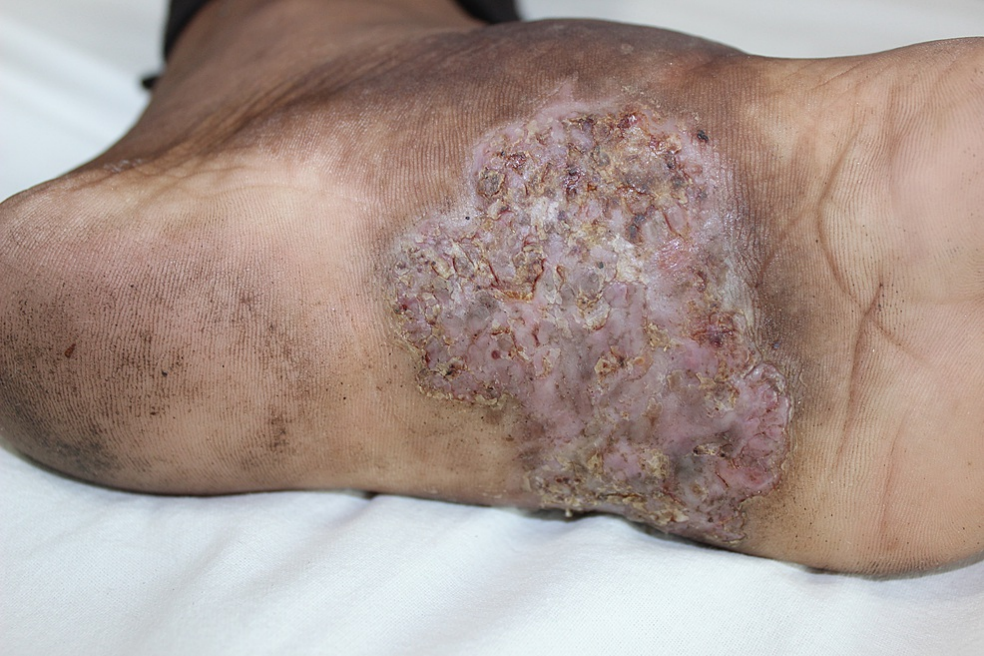
Well defined macerated erythematous plaque on the left sole with crusting and fissuring at places.

Seventeen-year-old male presented with involvement of left sole in the form of firm erythematous plaque for two years. The lesion was covered with scales at few places and had a macerated look at other sites (Figure 1).

**Figure 2:**
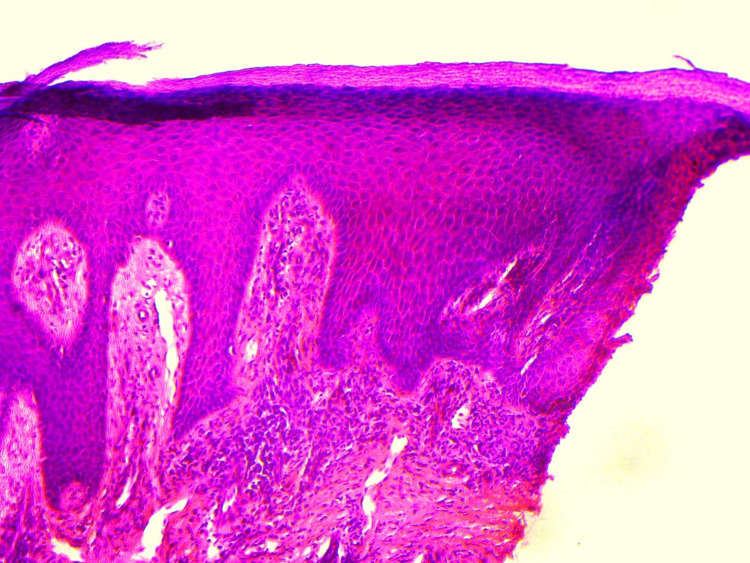
(H&E, 200×) Histopathology shows band like upper dermal infiltrate, focal vacuolar basal cell layer degeneration, and occasional necrotic keratinocytes.

The pain was minimal, mostly associated with walking discomfort. The rest of the mucocutaneous and scalp examination was normal. He had tried over-the-counter medications with no significant improvement. There was no history of preceding trauma and discharge from the lesions. The differentials of endogenous dermatitis, lichen planus, subcutaneous fungal infection, and squamous cell carcinoma were kept and the lesion was subjected to punch biopsy of the skin. The histopathology report showed dense band-like infiltrate in the papillary dermis, focal vacuolar degeneration of the basal cell layer, and occasional necrotic keratinocytes (Figure 2).

Based on clinical and characteristic histopathological findings diagnosis of plantar lichen planus was kept and the patient was started on halobetasol propionate 0.05%. He had 30-40% improvement in the following four to six weeks but unfortunately was then lost to follow-up.

## Discussion

Lichen planus can occur at any age but it presents highest in age groups 30-60 and its incidence in India is about 0.38% [1]. The severity of the disease may vary from few papules to acute generalized disease. The lesions of classic LP involve the flexor surface of the body, most commonly arms and legs, while the face and scalp are usually spared. Oral involvement is also common with visible Wickham's striae [5]. Soles are more involved than the palms in palmoplantar LP [6]. The characteristic lesion of classic LP is polygonal, plain topped, purple papule associated often with pruritus. Palmoplantar LP in contrast to classic LP presents with large-sized hyperkeratotic papules that often aggregate to form semi-translucent plaques [1]. The differential diagnosis of palmoplantar LP includes psoriasis, tinea manuum infection, lichenoid drug reaction, subcutaneous fungal infections, and keratoacanthoma [3]. The histopathology is almost similar for all the variants. It shows hyperkeratosis, wedge-shaped hypergranulosis, acanthosis, degeneration of the basal cell layer. Biopsy often shows civatte bodies and Max Joseph space. Saw-tooth rate ridges are another peculiar histopathological feature of LP [7].

Karakatsanis et al. described a variant of PPLP with umbilicated papules which showed resistance to topical corticosteroids and emollients. The patient was successfully treated with cyclosporine, an immunomodulating drug, with an initial dose of 3.5 mg/kg/day continued for four weeks. The dose was subsequently tapered for four weeks with the patient showing signs of clinical improvement [8]. Miotti et al. described a case of plantar erosive lichen planus, irresponsive to traditionally available treatment options like topical steroid preparations. The patient was then started on methotrexate, which resulted in the resolution of the ulcer without improvement of pain. To improve the life quality, the patient underwent a surgical treatment that included a combination of meshed split-thickness autologous skin graft and autologous skin micrograft [9].

Literature also has a mention of a case of PPLP that was successfully treated with acitretin. Initially, it was started at a dose of 35 mg and increased gradually up to 50 mg daily until 12 weeks after which the dose was tapered. The only significant side effect noted was mild cheilitis [10].

## Conclusions

Lichen planus affecting the palmo-plantar area may have a quite different clinical presentation, with minimal itching. It can be an isolated involvement of palms or soles making the diagnosis more difficult. Treating LP at these sites can be challenging and more insight is needed regarding treatment protocol or outcome of such cases.
